# Toward a working definition of ketogenic diet resistance in GLUT1 deficiency syndrome

**DOI:** 10.1002/epd2.70107

**Published:** 2025-09-30

**Authors:** Raffaele Falsaperla, Vincenzo Sortino, Gerhard Josef Kluger, Thomas Herberhold, Andrea Rüegger, Pasquale Striano, Martino Ruggieri, Joerg Klepper, Georgia Ramantani

**Affiliations:** ^1^ Department of Medical Sciences‐Pediatric Section University of Ferrara Ferrara Italy; ^2^ Unit of Pediatrics and Pediatric Emergency Azienda Ospedaliero‐Universitaria Policlinico “Rodolico‐San Marco,” San Marco Hospital, University of Catania Catania Italy; ^3^ Department of Pediatrics Institute of Rehabilitation, Transition and Palliation of Neurologically Ill Children, Paracelsus Medical University Salzburg Austria; ^4^ Clinic for Neuropediatrics and Neurorehabilitation Epilepsy Center for Children and Adolescents, Schön Klinik Vogtareuth Vogtareuth Germany; ^5^ Department of Neuropediatrics University Children's Hospital Zurich Zurich Switzerland; ^6^ Pediatric Neurology and Muscular Diseases Unit IRCCS Istituto Giannina Gaslini, Full Member of ERN‐Epicare Genoa Italy; ^7^ Department of Neurosciences, Rehabilitation, Ophthalmology, Genetics, Maternal and Child Health University of Genova Genoa Italy; ^8^ Unit of Rare Diseases of the Nervous System in Childhood, Department of Clinical and Experimental Medicine, Section of Pediatrics and Child Neuropsychiatry University of Catania Catania Italy; ^9^ Klinikum Aschaffenburg‐Alzenau Aschaffenburg Germany; ^10^ University of Zurich Zurich Switzerland

**Keywords:** GLUT1DS, ketogenic diet resistance, metabolic disease

## Abstract

**Objective:**

The ketogenic diet (KD) is the standard treatment for glucose transporter type 1 deficiency syndrome (GLUT1‐DS), typically yielding seizure reduction and cognitive/motor gains. However, a small subset of patients shows limited or no clinical benefit. This phenomenon, referred to as “KD resistance,” remains poorly understood and inconsistently defined. We propose a working definition, outline evaluation domains, summarize candidate mechanisms, and indicate management steps.

**Methods:**

Narrative review of published evidence and expert opinion across clinical, biochemical, EEG, and adherence domains.

**Results:**

KD resistance may be considered when all are present: (1) confirmed therapeutic ketosis (serial blood β‐hydroxybutyrate ≥2.0–2.5 mmol/L on repeated measurements); (2) adequate dietary adherence verified by dietetic assessment and, when available, validated tools; (3) sufficient trial duration (≥3 months; longer for primarily cognitive/motor goals); and (4) lack of meaningful improvement on symptom‐relevant standardized measures. EEG interictal epileptiform discharge burden can be used as an adjunct marker but is not required. KD resistance may involve several dimensions: failure to achieve therapeutic ketosis, lack of symptom improvement despite confirmed ketosis, or challenges with adherence that limit efficacy. Possible contributing factors include genotypic variability in SLC2A1, mitochondrial dysfunction, impaired blood–brain barrier transport, hormonal influences, and epigenetic regulation. We outline a multidomain evaluation framework with suggested metrics, summarize candidate mechanisms (ketone transport/utilization, mitochondrial function, neurotransmission, ion channels/neuromodulators, inflammation/oxidative stress, epigenetic regulation), and indicate when to introduce a KD‐compatible anti‐seizure medication.

**Significance:**

The proposed definition and framework standardize terminology and reporting, guide decisions for suboptimal responders, and set priorities for multicenter validation.

**Key points**

- Working definition of KD resistance in GLUT1‐DS with four core elements.
- Elements: therapeutic ketosis, adherence, ≥3‐month trial, lack of meaningful improvement.
- EEG IED burden may serve as an adjunct marker, not required for classification.
- Multidomain framework: clinical, cognitive, EEG, biochemical, duration, adherence.
- Guidance on when to add KD‐compatible ASM and how to monitor.

## 1 INTRODUCTION

Glucose transporter type 1 deficiency syndrome (GLUT1‐DS) is a rare metabolic disorder caused by pathogenic variants in the *SLC2A1* gene, which encodes the GLUT1 protein responsible for glucose transport across the blood–brain barrier (BBB) and astrocyte membranes.^1^ Impaired glucose transport in GLUT1‐DS leads to chronic brain energy deficiency, resulting in a range of age‐dependent neurological symptoms including drug‐resistant epilepsy, movement disorders, and developmental delay.^2^, ^3^, ^4^ Generalized seizures are the most frequent type, although other seizure types may also occur. In infancy, the combination of epilepsy with movement disorders should raise suspicion for GLUT1‐DS. Movement disorders can range from paroxysmal eye‐head movements in infancy to episodic ataxia, dystonia, or transient weakness later in childhood. Cognitive impairment is frequently observed, with visuospatial and visuomotor abilities typically more impaired than verbal skills,^5^ although verbal deficits may also be significant and should be considered when interpreting cognitive outcomes.

The ketogenic diet (KD) is the established first‐line therapy for GLUT1‐DS. This high‐fat, low‐carbohydrate diet with adequate protein generates ketones, mimicking the metabolic effects of fasting.^6^ These ketones bypass the glucose transport defect via monocarboxylate transporters (MCTs), providing an alternative energy source.^2^, ^7^, ^8^ KD effectively reduces seizure frequency, achieving seizure freedom in 67–90% of cases and a significant reduction in 80–100%. Earlier initiation, particularly in the first postnatal months, yields better outcomes,^2^, ^9^, ^10^ especially for non‐epileptic symptoms.^11^ Delayed diagnosis or treatment may allow persistent interictal epileptiform discharges (IEDs) to develop; in the immature brain, such activity may indicate secondary network activation and could reduce responsiveness to KD.^12^ Beyond seizure control,^13^, ^14^, ^15^ KD improves cognitive performance^4^—including total and verbal IQ,^4^ expressive and receptive language, sensorimotor speed, and attention.^16^ KD also alleviates movement disorders, particularly in older children and in presentations marked by developmental delay and movement disorders without prominent seizures.

Despite the established benefits of KD in GLUT1‐DS, a small number of patients show limited or no response, a phenomenon referred to as “KD resistance.” This remains poorly defined and insufficiently studied, partly due to limited published data, inconsistent definitions, and variability in clinical presentation and outcome reporting across studies. The rarity of GLUT1‐DS further complicates the identification and systematic evaluation of such cases.^17^, ^18^ The incidence or prevalence of KD resistance in GLUT1‐DS has not been established. Published evidence consists of small series and a focused literature review that identified only five published non‐responder cases and found no reliable predictors^18^; a single‐center cohort reported 7 KDT failures among 70 patients over a decade,^17^ but definitions varied, limiting generalizability. Together, these gaps support the need for standardized criteria and prospective multicenter reporting.^16^, ^17^ Addressing this gap is important for improving treatment decisions and outcomes in patients who do not respond as expected. Clear definitions and consistent reporting may also help identify mechanisms underlying KD resistance and support the development of more targeted management strategies. This paper focuses on KD resistance in GLUT1‐DS, emphasizes the need for a standardized definition, and outlines possible contributing factors and research priorities.

## 2 THE IMPORTANCE OF DEFINING KD RESISTANCE

“KD resistance” broadly refers to the failure of KD to achieve anticipated clinical benefits. However, the lack of a standardized definition leads to inconsistencies in how the term is used across clinical and research settings.^1^, ^19^ Current interpretations vary, including failure to achieve therapeutic ketosis (inefficacy), persistence or worsening of symptoms despite adequate ketosis (resistance), and treatment discontinuation or limited benefit due to poor adherence or side effects. This variability, combined with the challenges of assessing adherence based on self‐report, complicates the identification, evaluation, and management of affected patients.^17^, ^18^

A clear and practical definition of KD resistance is important for several reasons. First, it provides a framework for clinical decision‐making, supporting the identification of patients who may benefit from adjustments to the standard KD protocol, alternative therapies, or further diagnostic evaluations. Second, it enables more consistent data collection and comparison across research efforts, facilitating the identification of patterns, mechanisms, and potential interventions. Finally, establishing a shared terminology can improve interdisciplinary communication and coordination, particularly between neurologists, dietitians, and geneticists involved in the management of GLUT1‐DS. In addition, some individuals may show persistent or fluctuating IED burden on EEG despite good clinical seizure control. Given possible longer‐term effects on cognition, language, and motor function, KD response is best interpreted across multiple domains rather than by seizure outcomes alone.^12^

## 3 PROPOSED DIMENSIONS OF KD RESISTANCE

Understanding the factors contributing to KD resistance is a necessary step toward improving clinical care and advancing research in GLUT1‐DS. Given the complexity of this phenomenon, a structured approach that considers biochemical, dietary, and clinical dimensions may help guide treatment decisions and support more consistent reporting. Systematic evaluation of these domains could assist clinicians in identifying subgroups of patients who may benefit from tailored interventions.

One important challenge is determining what constitutes meaningful clinical improvement. In some patients, seizure reduction may be the primary goal, while in others, improvements in cognition or movement disorders may take precedence. Definitions of treatment response should therefore be individualized and supported by standardized tools that assess seizure activity, motor function, and cognitive performance before and during KD therapy. Verbal impairment, which may be underestimated in some individuals, can also affect the interpretation of cognitive testing and should be taken into account when evaluating KD response.^20^ When available, EEG assessment of IED burden—including in patients with good clinical seizure control—may complement clinical and biochemical measures as an adjunct marker of network instability relevant to KD response, though evidence remains limited.^12^

Biochemical markers are another essential component. Difficulty achieving or maintaining therapeutic ketosis may contribute to poor clinical response. For this reason, defining threshold values for markers such as blood beta‐hydroxybutyrate is important for evaluating whether adequate ketosis has been established.

The duration of KD treatment also influences the interpretation of response. Prematurely discontinuing the diet may lead to the misclassification of potential responders as resistant. A minimum trial period is essential to ensure that the diet's full impact is adequately evaluated before making clinical decisions. Furthermore, consensus on study protocols and diagnostic tools is critical to assess motor and cognitive resistance. This is particularly challenging, as cognitive assessments are age‐dependent and need to be repeated at 12‐ to 18‐month intervals to yield valid results.

Dietary adherence also plays a key role in distinguishing true resistance from non‐adherence (rather than “noncompliance”). The KD requires strict macronutrient ratios that are challenging to maintain, and inadequate adherence can mimic resistance. Thorough assessments of dietary compliance are necessary to rule out non‐compliance as a confounding factor in suboptimal outcomes. For consistency, we use the term “adherence” to reflect the degree to which patients and families are able to follow prescribed dietary regimens in daily life. Validated questionnaires developed in different countries can support this process by providing structured tools to assess adherence across behavioral, practical, and nutritional domains.^21^, ^22^, ^23^ Their routine use may help standardize adherence evaluation and distinguish implementation issues from true treatment resistance.

Lastly, developing a standardized evaluation framework is fundamental for objectively assessing KD efficacy. Such a framework should incorporate multiple dimensions, including biochemical markers, seizure frequency, cognitive and motor function, and overall quality of life. In the long term, this may support efforts to identify patterns of true resistance and inform alternative strategies for those not responding to standard KD protocols.

These proposed dimensions may serve as a starting point for improving the characterization of KD resistance and informing more individualized management approaches in GLUT1‐DS (see Table 1).

**TABLE 1:** Proposed dimensions of ketogenic diet resistance in glucose transporter type 1 deficiency syndrome.

Dimension	Considerations	Measures
Clinical outcome	Define individualized goals based on the patient's primary symptoms	Seizure diaries, standardized motor scales, developmental assessments
Cognitive testing	Verbal deficits may confound testing; cognitive change may emerge slowly	Age‐appropriate cognitive tests; repeated evaluations at 12‐ to 18‐month intervals
EEG/IED burden	IEDs may persist despite seizure control and indicate network instability; adjunctive	Baseline and follow‐up EEG; quantify IED frequency (e.g., spikes/hour, spike–wave index); document sleep vs. wake
Biochemical ketosis	Sustained ketosis is necessary to evaluate dietary efficacy	Target: β‐hydroxybutyrate ≥2.0–2.5 mmol/L
Duration of KD trial	Avoid premature discontinuation that may misclassify delayed responders	Minimum 3‐month trial; longer durations for cognitive or motor evaluation
Adherence	Suboptimal response may reflect inadequate adherence or unrecognized challenges	Dietitian assessment, food logs, validated adherence questionnaires
Assessment tools	Consistent tools improve interpretation and cross‐patient comparison	Seizure diaries, cognitive/motor scales, patient‐reported outcomes
Evaluation framework	Integration of clinical, biochemical, and functional domains improves reliability	Combine clinical, biochemical, developmental, and quality‐of‐life domains

Abbreviations: IED, interictal epileptic discharges; KD, ketogenic diet.

## 4 PROPOSED WORKING DEFINITION OF KD RESISTANCE

In this context, “ketogenic diet” refers to any form of ketogenic dietary therapy that achieves and sustains ketosis, including the classical KD, modified Atkins diet (MAD), and other structured protocols. Based on the dimensions outlined above, we propose a working definition of KD resistance in GLUT1‐DS to support consistent terminology and facilitate further research. KD resistance may be considered present when a patient shows persistent clinical symptoms—such as seizures, movement disorders, or stagnation in cognitive development—despite documented therapeutic ketosis and adequate dietary adherence over a sufficient trial period.

More specifically, we suggest that all of the following elements be considered:

1. Confirmed therapeutic ketosis, defined as sustained blood beta‐hydroxybutyrate levels ≥2.0–2.5 mmol/L, interpreted in the clinical context and with consideration of age‐related metabolic variability.
2. Adequate adherence to the prescribed KD, verified through clinical dietetic assessment and caregiver reports, is supported where possible by food records or indirect biochemical indicators.
3. Trial duration: ≥3 months of continuous KD with documented therapeutic ketosis; extend to ~6 months for primarily cognitive/motor goals or until the next age‐appropriate assessment (typically 12–18 months). Early discontinuation due to intolerance or inability to achieve ketosis should not be labeled resistance.
4. Lack of meaningful clinical improvement, based on standardized or clearly defined measures relevant to the patient's primary symptoms (e.g., seizure diaries, developmental or motor scales, clinical observation); IED burden may be used as an adjunctive marker where available but is not required for classification.

This working definition is intended as a conceptual tool, not a diagnostic criterion. It may help distinguish true resistance from delayed responders, suboptimal implementation, or non‐adherence. We acknowledge the limitations of current evidence and emphasize that further validation in prospective studies is needed.

## 5 POTENTIAL MECHANISMS OF KETOGENIC RESISTANCE IN GLUT1‐DS

The mechanisms contributing to reduced KD efficacy in GLUT1 deficiency syndrome (GLUT1‐DS) are likely multifactorial, reflecting the underlying complexity of the disorder. A better understanding of these mechanisms may help improve treatment strategies for patients who do not respond as expected to dietary therapy.

Proposed mechanisms through which ketone bodies may influence brain excitability and network stability include: (i) provision of alternative fuel via monocarboxylate transporter–mediated uptake and mitochondrial ATP generation; (ii) neurotransmission effects (enhanced GABAergic tone and/or reduced glutamatergic release); (iii) ion channel/neuromodulatory effects (e.g., K_ATP, adenosine A1, HCN)^24^; (iv) improved mitochondrial function/redox balance; (v) anti‐inflammatory signaling (e.g., NLRP3 attenuation); and (vi) epigenetic regulation (e.g., histone deacetylase inhibition).^25^, ^26^ Disruption at any of these steps—transport, mitochondrial function, signaling, or transcriptional regulation—could limit response.

Genotypic variability in SLC2A1,^3^ the gene encoding the GLUT1 protein, appears to influence treatment response. Different mutation types may result in varying levels of residual glucose transport activity. For example, missense mutations may allow partial function of the GLUT1 transporter, reducing the brain's reliance on ketones and potentially attenuating the effects of KD. In some cases, additional pathogenic or likely pathogenic variants may also contribute to atypical presentations or reduced KD response. While GLUT1‐DS is defined by SLC2A1 mutations, exome or panel‐based testing may uncover other relevant genes affecting energy metabolism, transporter function, or mitochondrial activity. Comprehensive genetic evaluation is recommended in cases with incomplete response, particularly when clinical features extend beyond the typical GLUT1‐DS phenotype.

Disruption of metabolic pathways may also play a role.^2^ Secondary mitochondrial dysfunction or deficiencies in enzymes essential for ketone metabolism—such as succinyl‐CoA:3‐ketoacid transferase (SCOT) or acetoacetyl‐CoA thiolase—may impair cerebral ketone utilization, even when ketosis is achieved.

Structural or functional alterations in the BBB can also contribute to KD resistance.^27^ The reduced expression or activity of monocarboxylate transporters (MCTs), which are essential for transporting ketones across the BBB, may limit the availability of ketones to the brain, even in the presence of adequate peripheral ketosis.

Neuroinflammation and oxidative stress may further exacerbate energy deficits.^28^ Neuroinflammation and elevated levels of reactive oxygen species (ROS) can disrupt mitochondrial function and damage critical components of oxidative phosphorylation. These changes can compromise the brain's ability to utilize ketones, adding another layer of resistance. Elevated ROS have also been linked to increased seizure‐induced neuronal death, suggesting that failure to mitigate oxidative stress may limit the neuroprotective potential of KD.^26^

Epigenetic and regulatory factors also warrant attention.^29^ Modifications such as hypermethylation of promoters for genes related to ketone metabolism may suppress their expression, thereby limiting the brain's ability to metabolize ketones effectively. Broader dysregulation of genes involved in energy metabolism may similarly impair therapeutic outcomes.

Interestingly, sex‐based differences have emerged as a potential contributor to KD resistance.^30^ Documented cases of apparent KD resistance in GLUT1‐DS have more often been reported in female patients, suggesting hormonal or sex‐specific genetic influences. Fluctuations in hormones like estrogen and progesterone, particularly during the menstrual cycle, could modulate mitochondrial function or ketone metabolism. Additionally, sex‐specific epigenetic patterns, such as differential methylation or X‐linked modifiers, may affect the expression of genes critical for ketone utilization and glucose transport.

Finally, dietary and lifestyle factors significantly impact KD efficacy.^3^ Suboptimal energy intake, macronutrient imbalances, gastrointestinal dysfunction, and concurrent medications such as valproic acid may interfere with ketone production, absorption, or utilization. These factors can sometimes mimic true resistance or exacerbate underlying biological mechanisms.

Clarifying the relative contribution of these mechanisms will require systematic investigation in larger, well‐characterized patient cohorts. Prospective, multicenter studies integrating genetic, biochemical, dietary, and clinical data will be essential to better characterize KD resistance and inform individualized treatment approaches.

## 6 TOWARD A UNIFIED APPROACH

A standardized definition of KD resistance in GLUT1‐DS is essential for advancing both clinical practice and research. Collaborative efforts among specialists in neurology, metabolism, and dietetics are needed to develop shared criteria that can support evidence‐based decision‐making. Initiatives such as those within the Network for Therapy in Rare Epilepsies (NETRE)^23^ framework are important for gathering prospective data to identify genotypic and phenotypic factors associated with KD resistance in larger patient cohorts.

Patient‐centered approaches are also important. In this context, the use of patient‐reported outcome measures (PROMs) can help capture aspects of quality of life that are not always reflected in clinical or biochemical data.^25^ Engaging families in discussions about treatment goals, expectations, and adherence strategies can improve KD implementation and outcomes. For patients identified as resistant to KD, early recognition could facilitate adjunctive therapies, including medium‐chain triglycerides or pharmacological interventions targeting ketone metabolism.

After an adequate KD trial (e.g., ≥3 months with sustained therapeutic ketosis and verified adherence), introduce a KD‐compatible ASM if <50% seizure reduction or disabling events persist; earlier treatment is appropriate for status epilepticus, frequent clusters, or clear deterioration. Preferred first add‐ons include broad‐spectrum agents with good KD compatibility such as levetiracetam or clobazam; ethosuximide when absence seizures predominate, and lamotrigine by seizure type. Topiramate or zonisamide can be used selectively with monitoring for carbonic‐anhydrase–related effects (metabolic acidosis, nephrolithiasis). Generally, avoid valproate due to interference with ketosis; if used, monitor ketones and metabolic tolerance. Minimize polypharmacy and revisit de‐escalation once stabilized.

## 7 CONCLUSION

KD resistance in GLUT1‐DS should be considered when all of the following are present: (i) confirmed therapeutic ketosis (serial blood β‐hydroxybutyrate ≥2.0–2.5 mmol/L, interpreted in context); (ii) adequate adherence to the prescribed KD (dietetic assessment, with validated questionnaires when available); (iii) ≥3 months of continuous KD (longer for primarily cognitive/motor goals); and (iv) lack of meaningful clinical improvement on symptom‐relevant standardized measures. IED burden on EEG may be used as an adjunct marker of network instability but is not required for classification.

## FUNDING INFORMATION

We thank the National Science Foundation (SNSF: 208184) and the Anna Mueller Grocholski Foundation (to G.R.) for funding. The funders had no role in the design or analysis of the study.

## CONFLICT OF INTEREST STATEMENT

None of the authors have any conflict of interest to disclose.

8

**Test yourself**

1. Which of the following elements is required for considering ketogenic diet (KD) resistance in GLUT1 deficiency syndrome?
  - EEG evidence of persistent interictal epileptiform discharges
  - Confirmed therapeutic ketosis (β‐hydroxybutyrate ≥2.0–2.5 mmol/L)
  - Treatment duration of at least 6 weeks
  - Presence of a pathogenic SLC2A1 mutation
2. According to the proposed working definition, what is the minimum recommended duration of a KD trial before classifying a patient as resistant (when seizure control is the main treatment goal)?
  - 1 month
  - 3 months
  - 6 months
  - 12 months
3. Which of the following is not considered a potential mechanism of KD resistance in GLUT1 deficiency syndrome?
  - Impaired ketone transport across the blood–brain barrier
  - Mitochondrial dysfunction
  - Increased oxidative stress
  - Enhanced glucose transport via GLUT1 upregulation

*Answers may be found in the* *supporting information*

## Supporting information


Appendix S1.

## Data Availability

Data sharing not applicable to this article as no datasets were generated or analysed during the current study.

## References

[1] Klepper J , Leiendecker B . GLUT1 deficiency syndrome – 2007 update. Dev Med Child Neurol. 2007;49(9):707–716. 17718830 10.1111/j.1469-8749.2007.00707.x

[2] Leen WG , Klepper J , Verbeek MM , Leferink M , Hofste T , van Engelen BG , et al. Glucose transporter‐1 deficiency syndrome: the expanding clinical and genetic spectrum of a treatable disorder. Brain. 2010;133(Pt 3):655–670. 20129935 10.1093/brain/awp336

[3] Wang D , Pascual JM , De Vivo D . Glucose transporter type 1 deficiency syndrome. In: Adam MP , Feldman J , Mirzaa GM , Pagon RA , Wallace SE , Amemiya A , editors. GeneReviews®. Seattle, WA: University of Washington, Seattle; 1993. Available from: http://www.ncbi.nlm.nih.gov/books/NBK1430/ 20301603

[4] De Giorgis V , Masnada S , Varesio C , Chiappedi MA , Zanaboni M , Pasca L , et al. Overall cognitive profiles in patients with GLUT1 deficiency syndrome. Brain Behav. 2019;9(3):e01224. 30714351 10.1002/brb3.1224 PMC6422708

[5] Klepper J , Akman C , Armeno M , Auvin S , Cervenka M , Cross HJ , et al. Glut1 deficiency syndrome (Glut1DS): state of the art in 2020 and recommendations of the international Glut1DS study group. Epilepsia Open. 2020;5(3):354–365. 32913944 10.1002/epi4.12414 PMC7469861

[6] Wheless JW . History of the ketogenic diet. Epilepsia. 2008;49(Suppl 8):3–5. 10.1111/j.1528-1167.2008.01821.x 19049574

[7] Ramm‐Pettersen A , Nakken KO , Skogseid IM , Randby H , Skei EB , Bindoff LA , et al. Good outcome in patients with early dietary treatment of GLUT‐1 deficiency syndrome: results from a retrospective Norwegian study. Dev Med Child Neurol. 2013;55(5):440–447. 23448551 10.1111/dmcn.12096

[8] Kass HR , Winesett SP , Bessone SK , Turner Z , Kossoff EH . Use of dietary therapies amongst patients with GLUT1 deficiency syndrome. Seizure. 2016;35:83–87. 26803281 10.1016/j.seizure.2016.01.011

[9] Leary LD , Wang D , Nordli DR , Engelstad K , De Vivo DC . Seizure characterization and electroencephalographic features in Glut‐1 deficiency syndrome. Epilepsia. 2003;44(5):701–707. 12752470 10.1046/j.1528-1157.2003.05302.x

[10] Klepper J , Scheffer H , Leiendecker B , Gertsen E , Binder S , Leferink M , et al. Seizure control and acceptance of the ketogenic diet in GLUT1 deficiency syndrome: a 2‐ to 5‐year follow‐up of 15 children enrolled prospectively. Neuropediatrics. 2005;36(5):302–308. 16217704 10.1055/s-2005-872843

[11] Alter AS , Engelstad K , Hinton VJ , Montes J , Pearson TS , Akman CI , et al. Long‐term clinical course of Glut1 deficiency syndrome. J Child Neurol. 2015;30(2):160–169. 24789115 10.1177/0883073814531822

[12] Toro‐Perez J , Healy S , Sell E , Bulusu S , Whiting S . Role of EEG as a monitoring tool in patients with glucose transporter type I deficiency syndrome (GLUT1‐DS) on ketogenic diet. Epileptic Disord. 2023;25(3):360–370. 37070482 10.1002/epd2.20063

[13] Sandu C , Burloiu CM , Barca DG , Magureanu SA , Craiu DC . Ketogenic diet in patients with GLUT1 deficiency syndrome. Maedica. 2019;14(2):93–97. 31523287 10.26574/maedica.2019.14.2.93 PMC6709387

[14] Klepper J . GLUT1 deficiency syndrome in clinical practice. Epilepsy Res. 2012;100(3):272–277. 21382692 10.1016/j.eplepsyres.2011.02.007

[15] Ito Y , Oguni H , Ito S , Oguni M , Osawa M . A modified Atkins diet is promising as a treatment for glucose transporter type 1 deficiency syndrome. Dev Med Child Neurol. 2011;53(7):658–663. 21501156 10.1111/j.1469-8749.2011.03961.x

[16] Ramm‐Pettersen A , Stabell KE , Nakken KO , Selmer KK . Does ketogenic diet improve cognitive function in patients with GLUT1‐DS? A 6‐ to 17‐month follow‐up study. Epilepsy Behav. 2014;39:111–115. 25240122 10.1016/j.yebeh.2014.08.015

[17] Bekker YAC , Lambrechts DA , Verhoeven JS , van Boxtel J , Troost C , Kamsteeg E‐J , et al. Failure of ketogenic diet therapy in GLUT1 deficiency syndrome. Eur J Paediatr Neurol. 2019;23(3):404–409. 30885501 10.1016/j.ejpn.2019.02.012

[18] Falsaperla R , Sortino V , Kluger GJ , Herberhold T , Rüegger A , Striano P , et al. Exploring ketogenic diet resistance in glucose transporter type 1 deficiency syndrome: a comprehensive review and critical appraisal. Epilepsia Open. 2025;10(1):31–39. 39641282 10.1002/epi4.13110 PMC11803274

[19] Kossoff EH , Zupec‐Kania BA , Auvin S , Ballaban‐Gil KR , Christina Bergqvist AG , Blackford R , et al. Optimal clinical management of children receiving dietary therapies for epilepsy: updated recommendations of the international ketogenic diet study group. Epilepsia Open. 2018;3(2):175–192. 29881797 10.1002/epi4.12225 PMC5983110

[20] Corradini M , Zanaboni MP , Varesio C , Celario M , Capelli E , Giudice C , et al. GLUT1DS focus on dysarthria. Eur J Paediatr Neurol. 2024;51:62–70. 38851145 10.1016/j.ejpn.2024.05.010

[21] Lopes Neri L d C , Guglielmetti M , De Giorgis V , Pasca L , Zanaboni MP , Trentani C , et al. Validation of an Italian questionnaire of adherence to the ketogenic dietary therapies: iKetoCheck. Foods. 2023;12(17):3214. 37685147 10.3390/foods12173214 PMC10486753

[22] Neri L , Sampaio LP . Validation of ketogenic diet adherence questionnaire: keto‐check. Arq Neuropsiquiatr. 2022;80(8):794–801. 36252587 10.1055/s-0042-1755343 PMC9703890

[23] Lopes Neri L d C , Ferraro AA , Guglielmetti M , Fiorini S , Sampaio LP d B , Tagliabue A , et al. Factor analysis of the Brazilian questionnaire on adherence to ketogenic dietary therapy: keto‐check. Nutrients. 2023;15(17):3673. 37686705 10.3390/nu15173673 PMC10489998

[24] Wells J , Swaminathan A , Paseka J , Hanson C . Efficacy and safety of a ketogenic diet in children and adolescents with refractory epilepsy‐a review. Nutrients. 2020;12(6):1809. 32560503 10.3390/nu12061809 PMC7353240

[25] DiFrancesco JC , Ragona F , Murano C , Frosio A , Melgari D , Binda A , et al. A novel de novo HCN2 loss‐of‐function variant causing developmental and epileptic encephalopathy treated with a ketogenic diet. Epilepsia. 2023;64(12):e222–e228. 37746765 10.1111/epi.17777

[26] Youngson NA , Morris MJ , Ballard JWO . The mechanisms mediating the antiepileptic effects of the ketogenic diet, and potential opportunities for improvement with metabolism‐altering drugs. Seizure. 2017;52:15–19. 28941398 10.1016/j.seizure.2017.09.005

[27] Xu W , Borges K . Case for supporting astrocyte energetics in glucose transporter 1 deficiency syndrome. Epilepsia. 2024;65(8):2213–2226. 38767952 10.1111/epi.18013

[28] Tang M , Park SH , Petri S , Yu H , Rueda CB , Abel ED , et al. An early endothelial cell‐specific requirement for Glut1 is revealed in Glut1 deficiency syndrome model mice. JCI Insight. 2021;6(3):e145789. 33351789 10.1172/jci.insight.145789 PMC7934852

[29] Olivotto S , Duse A , Bova SM , Leonardi V , Biganzoli E , Milanese A , et al. Glut1 deficiency syndrome throughout life: clinical phenotypes, intelligence, life achievements and quality of life in familial cases. Orphanet J Rare Dis. 2022;17(1):365. 36153584 10.1186/s13023-022-02513-4 PMC9509642

[30] Cervenka MC , Henry B , Nathan J , Wood S , Volek JS . Worldwide dietary therapies for adults with epilepsy and other disorders. J Child Neurol. 2013;28(8):1034–1040. 23670244 10.1177/0883073813488671

