# Supplementary material for: Toward a working definition of ketogenic diet resistance in GLUT1 deficiency syndrome
Source: Epileptic Disord. 2025 Sep 30;27(6):1247–54. doi: 10.1002/epd2.70107 (PMC12747689; doi:10.1002/epd2.70107)
Supplement: Supplementary file 1 — Appendix S1. [file EPD2-27-1247-s001.docx]

**TEST YOURSELF**

**Answers:**

**1. B**

**2. B**

**3. D**
